# More than half a century of evolutionary studies in *Rhodnius prolixus* Stål, 1859 (Hemiptera, Triatominae): revisiting and discussing old and new data on intra- and interspecific reproductive barriers

**DOI:** 10.1371/journal.pone.0335238

**Published:** 2025-10-31

**Authors:** Kaio Cesar Chaboli Alevi, Amanda Ravazi, Jader de Oliveira, Yago Visinho dos Reis, Isadora de Freitas Bittinelli, Luiza Maria Grzyb Delgado, Jociel Klleyton Santos Santana, Maria Tercília Vilela de Azeredo-Oliveira, João Aristeu da Rosa, Mauro Toledo Marrelli, Cleber Galvão

**Affiliations:** 1 Laboratory of Entomology in Public Health, Department of Epidemiology, Faculty of Public Health (FSP), University of Sao Paulo (USP), Sao Paulo, Brazil; 2 National and International Reference Laboratory on Triatomine Taxonomy, Oswaldo Cruz Institute (IOC), Oswaldo Cruz Foundation (FIOCRUZ), Rio de Janeiro, Brazil; 3 São Paulo State University (UNESP), Institute of Biosciences, Botucatu , Brazil; 4 Laboratory of Parasitology, São Paulo State University (UNESP), School of Pharmaceutical Sciences (FCFAR), Sao Paulo, Brazil; 5 Department of Entomology, National Museum of Natural History, Smithsonian Institution, Washington, DC, United States of America; 6 São Paulo State University (UNESP), Institute of Biosciences, Humanities and Exact Sciences (IBILCE), São José do Rio Preto, Brazil; Universidade Estadual Paulista: Universidade Estadual Paulista Julio de Mesquita Filho, BRAZIL

## Abstract

*Rhodnius* represents a paraphyletic group, being *R. prolixus* one of the most important domestic vectors of the Chagas disease. Several phenotypic identification problems, as well as divergences between classical and molecular taxonomy, have been reported. Furthermore, phylogenetic and phylogenomic studies demonstrated possible introgression events between *R. prolixus* and *R. robustus*. Based on the above, we revisited all the literature on hybridization involving *R. prolixus* and performed interspecific crosses between *R. prolixus* and other species of the *R. prolixus* group (*R. nasutus*, *R. neivai*, and *R. robustus*) to evaluate potential reproductive barriers and discuss taxonomic and evolutionary issues related to intra- and interspecific reproductive isolation. With the exception of the cross between *R. prolixus* females and *R. neivai* males, all other combinations resulted in hybrid offspring. Moreover, except for the cross between *R. prolixus* females and *R. robustus* males, all other combinations exhibited postzygotic barriers, including inviability, sterility and/or hybrid collapse. These results indicate that, in at least one direction, *R. nasutus*, *R. neivai*, and *R. robustus* are reproductively isolated from *R. prolixus*, confirming the specific status of the four taxa. Furthermore, based on the observed barriers, we suggest that introgression is unlikely between *R. prolixus* and *R. nasutus*, unlike *R. neivai* and *R. robustus*, which could exchange genetic material with *R. prolixus* through introgression, under natural conditions. Finally, we discuss all available literature on intra- and interspecific crosses of *R. prolixus*, demonstrating that *R. pictipes* and *R. neglectus* are also reproductively isolated from *R. prolixus*. Additionally, we highlight reproductive barriers observed between allopatric populations of *R. prolixus*, emphasizing the need for a phylogenomic study – including field-collected specimens sampled across the entire distribution of *R. prolixus* – to clarify evolutionary and taxonomic questions.

## Introduction

Chagas disease (CD) is a neglected vector-borne disease that affects six to seven million people worldwide [1], causing around 12,000 deaths per year and putting at risk of infection another 75 million people, particularly those livingin socially vulnerable conditions, such as areas close to vectors, reservoirs, or both [1,2]. Although there are other routes of infection (e.g., blood transfusion, organ transplantation, transplacental transmission, and consumption of contaminated food) [1,2], vector-borne transmission by triatomines remains the primary mode of infection, making vector control the main strategy for mitigating new cases of CD [1,2].

Chagas disease is caused by the protozoan *Trypanosoma cruzi* (Chagas, 1909) (Kinetoplastida, Trypanosomatidae) [3]. This protozoan exhibit significant genetic variability, classified into discrete typing units (DTUs) ranging from TcI to TcVI, plus TcBat [4], and infects more than 150 mammalian species [2] and, to date, one bird species [5]. In addition to vertebrate hosts, invertebrate hosts of the Triatominae subfamily also participate in the heteroxenous life cycle of *T. cruzi* [3]. Triatomines are hematophagous insects that have the habit of defecating/urinating during or shortly after a blood meal [1,3]. When feeding on infected hosts, they acquire the parasite and, once infected by *T. cruzi*, they release it in feces/urine, regardless of sex or stage of development [1].

Currently, 158 species of triatomines are known [6]. These insects are taxonomically classified into five tribes and 19 genera [6,7], with *Triatoma* Laporte, 1832, *Panstrongylus* Berg, 1879, and *Rhodnius* Stål, 1859 being the most epidemiologically significant [8]. The genus *Rhodnius* is a paraphyletic group composed of 19 species, being *R. prolixus* Stål, 1859 long considered one of the most important domestic vectors of the CD in northern South America and Central America [9,10].

It is believed that *R. prolixus* originated in South America and later spread to all Central American countries [11]. This exotic species was introduced into Central America in the early 20th century (1910) when various specimens were brought from a European university to El Salvador for research purposes but they accidentally escaped from a laboratory [11]. Following the implementation of the Initiative of the Countries of Central America and Mexico for the Control of Vector-borne and Transfusional Transmission and Medical Care for Chagas Disease (IPCAM), several countries were declared free of vector transmission by *R. prolixus* [11]. However, in 2019, specimens were captured in households in Mexico, highlighting the need for continuous monitoring [12].

From a systematic perspective, *R. prolixus* belongs to the *R. prolixus* group [6,8,13–18]. In addition to this species, *R. barretti* Abad-Franch, Palomeque & Monteiro, 2013, *R. dalessandroi* Carcavallo & Barreto, 1976, *R. domesticus* Neiva & Pinto, 1923, *R. marabaensis* Souza et al., 2016, *R. montenegrensis* Rosa et al., 2012, *R. nasutus* Stål, 1859, *R. neglectus* Lent, 1954, *R. neivai* Lent, 1953 and *R. robustus* Larrousse, 1927, as well as *Psammolestes* spp., are also part of this phylogenetically related species group [6,8,13–18]. Several challenges in phenotypic identification have been reported [19], along with discrepancies between classical and molecular taxonomy regarding *R. prolixus* and other species within the *R. prolixus* group [13].

Phylogenetic [15,20–22] and phylogenomic [13,23] studies have suggested possible introgression events between *R. prolixus* and *R. robustus*. Fitzpatrick et al. [20] associated these events with potential hybridization zones in Venezuela. Several authors have evaluated the hybridization capacity of these species under laboratory conditions, yielding contradictory results. Some studies indicate a total absence of reproductive barriers [24–27], while others report the presence of prezygotic [27] and postzygotic barriers [24,25]. These inconsistencies may be partly attributed to challenges in accurately identifying these species [13,19], potentially leading to the misclassification of other taxa as *R. prolixus* and *R. robustus*.

Furthermore, intraspecific crosses have been conducted, revealing intriguing reproductive patterns. Some populations of *R. prolixus* from Brazil, Venezuela, Honduras and Colombia exhibited postzygotic (inviability, sterility and/or collapse sterility) barriers [24,25]. The inviability – mortality of offspring before reaching adulthood – or sterility of first-generation hybrids (F1) could be result from genetic incompatibilities, loss of local adaptations, or disruption of co-adapted genes [28,29]. Already the hybrid collapse consists of the populational decline of hybrid lineage starting from second-generation hybrids (F2), due to high mortality rate or sterility, resulting from genetic dysregulation [30,31]. These findings suggest that these populations, initially identified as *R. prolixus*, are reproductively isolated and may, therefore, represent distinct species according to the biological species concept [30–33]).

In light of these observations, we revisited all the literature related to *R. prolixus* hybridization and performed interspecific crosses between *R. prolixus* and other species within the *R. prolixus* group (*R. nasutus*, *R. neivai*, and *R. robustus*) to evaluate the potential reproductive barriers and explore the taxonomic and evolutionary implications related to intra- and interspecific reproductive isolation.

## Methods

### Experimental crosses

**Fig 1:**
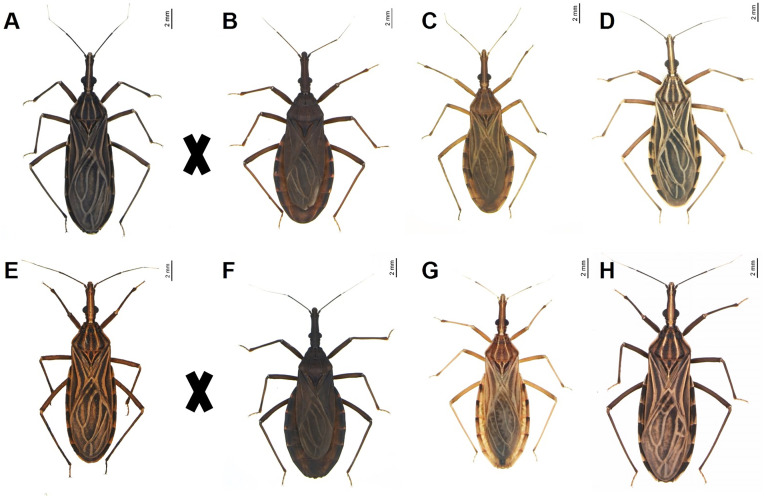
Species used in experimental crosses. **A.** *R. prolixus* ♀; **B.** *R. neivai* ♂; **C.** *R. nasutus* ♂; **D.** *R. robustus* ♂; **E.** *R. prolixus* ♂; **F.** *R. neivai* ♀; **G.** *R. nasutus* ♀; **H.** *R. robustus* ♀.

Reciprocal experimental crosses were conducted between *R. prolixus* (Colombia, Casanare, La Salina, peridomestic area) (Fig 1A and E) [34] with *R. neivai* (Venezuela, Carabobo, Valencia) (Fig 1B and F) [35], *R. nasutus* (Brazil, Rio Grande do Norte, Almino Afonso, peridomestic area) (Fig 1C and G) [36], and *R. robustus* (Peru, Lima) (Fig 1D and H) [37] (Table 1). The insects used in the experiment originated from colonies maintained at the Triatominae insectary of the School of Pharmaceutical Sciences, São Paulo State University (UNESP), Araraquara, São Paulo, Brazil. Species identification was performed using the dichotomous keys developed by Galvão [38].

**Table 1:** Experimental crosses performed between *R. prolixus* with *R. neivai*, *R. nasutus*, and **R. robustus*.*

	Experimental crosses		Number of eggs				Egg Fertility
	**Interspecific crosses**		**C1**	**C2**	**C3**	**Total**	
♀	*R. prolixus* x *R. neivai*	♂	110	115	143	368	00 (00%)
♀	*R. neivai* x *R. prolixus*^1^	♂	30	40	63	133	41 (31%)
♀	*R. nasutus* x *R. prolixus*^2^	♂	232	11	73	316	14 (04%)
♀	*R. prolixus* x *R. nasutus*^3^	♂	106	90	122	318	71 (22%)
♀	*R. robustus* x *R. prolixus*^4^	♂	175	156	291	622	447 (72%)
♀	*R. prolixus* x *R. robustus*^5^	♂	106	181	150	437	222 (51%)
	**Intercrosses**						
♀	Hybrid F1^1^ x Hybrid F1^1^	♂	167	49	96	312	293 (94%)
♀	Hybrid F2^1^ x Hybrid F2^1^	♂	41	22	61	124	50 (40%)
♀	Hybrid F3^1^ x Hybrid F3^1^	♂	34	16	42	92	00 (00%)
♀	Hybrid F1^2^ x Hybrid F1^2^	♂	82	146	161	309	00 (00%)
♀	Hybrid F1^3^ x Hybrid F1^3^	♂	175	134	172	481	00 (00%)
♀	Hybrid F1^4^ x Hybrid F1^4^	♂	650	397	294	1341	1285 (96%)
♀	Hybrid F2^4^ x Hybrid F2^4^	♂	111	228	274	613	305 (40%)
♀	Hybrid F3^4^ x Hybrid F3^4^	♂	77	101	98	276	00 (00%)
♀	Hybrid F1^5^ x Hybrid F1^5^	♂	287	292	274	853	827 (97%)
♀	Hybrid F2^5^ x Hybrid F2^5^	♂	184	254	83	521	401 (77%)
♀	Hybrid F3^5^ x Hybrid F3^5^	♂	103	256	25	384	322 (84%)
♀	Hybrid F4^5^ x Hybrid F4^5^	♂	204	117	130	451	393 (87%)
♀	Hybrid F5^5^ x Hybrid F5^5^	♂	20	15	21	55	25 (45%)
	**Control experiments**		**C1**	**C2**	**C3**	**Total**	
♀	*R. prolixus* x *R. prolixus*	♂	275	290	–	565	485 (86%)
♀	*R. neivai* x *R. neivai*	♂	90	87	–	177	92 (52%)
♀	*R. nasutus* x *R. nasutus*	♂	367	374	–	741	437 (59%)
♀	*R. robustus* x *R. obustus*	♂	173	194	–	367	269 (73%)

The experimental crosses were conducted in the Triatominae insectary, according to the methodologies of Mendonça et al. [31] and Reis et al. [39]: the insects were sexed as fifth-instar nymphs, and males and females were kept separately until they reached the adulthood in order to guarantee the virginity of the insects used in the crosses. For each cross, three couples from each set were placed separately in plastic jars (diameter 5 cm × height 10 cm) and maintained at room temperature (average of 24º C) with a relative humidity of 63% [40]. In addition, intraspecific crosses were also performed as group control (Table 1).

Eggs were collected weekly throughout the oviposition period, and the egg fertility rate was calculated. After the hybrids hatched, the development of first-instar nymphs was monitored weekly until adulthood to assess mortality rates. Once F1 nymphs reached adulthood, six new couples of F1 (three for each direction) were selected for intercrossing, with the same parameters described above used in the evaluation (Table 1). Additionally, F2 intercrosses were also conducted in both directions.

Crosses were carried out up to the third generation (F3) for *R. neivai* females and *R. prolixus* males, and *R. robustus* females and *R. prolixus* males, while *R. prolixus* females and *R. robustus* males crosses continued up to the fifth generation (F5) (Table 1). We clarify that for all quantitative data collected, the relative frequency was calculated.

### Cytogenetic analysis

Five adult male hybrids from each generation (F1-F5) were dissected, and their testes were removed and stored in a methanol: acetic acid solution (3:1). Slides were prepared by the cell-crushing technique, as described by Alevi et al. [41], and cytogenetic analyses were performed to characterize spermatogenesis, with an emphasis on the degree of pairing between the homologous chromosomes, using the lacto-acetic orcein technique [41,42]. The slides were examined under a light microscope (Jenamed; Carl Zeiss, Jena, Germany) coupled with a digital camera at 1000x magnification; AxioVision LE version 4.8 imaging software (Carl Zeiss) was used for analysis.

## Results and discussion

With the exception of the cross between *R. prolixus* females and *R. neivai* males that did not produce hybrids, all other combinations resulted in hybrid offspring (Table 1). The absence of hybrid hatching (in one or both directions) has been observed for intergeneric, such as, for example, *Rhodnius* with the genera *Triatoma* [43–45] and *Psammolestes* Bergroth, 1911 [46], and interspecific crosses, such as *R. prolixus* with *R. neglectus* [25], *R. prolixus* with *R. nasutus* [25], *R. prolixus* with *R. robustus* [25], and *R. pallescens* with *R. colombiensis* [47]. This evolutionary phenomenon is the result of the action of prezygotic reproductive barriers [30,32,33].

Among the different prezygotic isolation mechanisms, as temporal, ecological, habitat, behavioral, gametic and mechanical [30,32,33], Díaz et al. [47] suggested that mechanical isolation – due to incompatibilities between genitalia – was responsible for preventing the formation of hybrids between *R. pallescens* females with *R. colombiensis* males. Once interspecific copulations were observed between *R. prolixus* females and *R. neivai* males, we believe that mechanical isolation is not the mechanism responsible for reproductive inviability between these species. However, we emphasize that regardless of the barrier present, the non-production of hybrids under controlled laboratory conditions is a very important result, as it confirms the specific status of the parental species, based on the biological concept of species [30,32,33].

In addition to *R. neivai*, prezygotic isolation was also observed (in one or both directions) when *R. prolixus* from Colômbia, Brazil, Venezuela and/or Honduras was crossed with *R. nasutus* from Brazil [25], *R. robustus* from Venezuela [27], as well as *R. neglectus* from Brazil [24,25,43–45] (Table 2). However, although *R. prolixus* has been reported in several cases in Brazil [48–54], there are authors who consider that these records may have been misidentifications in relation to *R. neglectus*, *R. nasutus* and/or *R. robustus* and that this species is not present in Brazil [19,38].

**Table 2:** Intra- and interspecific experimental crosses performed with *R. prolixus.*

Experimental crosses	Prezygotic barrier	Poszygotic barrier			References
Interspecific crosses (*R. prolixus* x *Rhodnius* spp.)		Hybrid inviability	Hybrid sterility	Hybrid collapse	
***R. neivai***					
*R. prolixus* ♀ x *R. neivai* ♂	Present	–	–	–	This paper
*R. neivai* ♀ x *R. prolixus* ♂	Absent	–	–	Present	This paper
***R. pictip*e*s***					
*R. prolixus* ♀ x *R. pictip*e*s* ♂	Absent	Present	–	–	68
*R. pictip*e*s* ♀ x *R. prolixus* ♂	Absent	Present	–	–	68
***R. nasutus***					
*R. prolixus* ♀ x *R. nasutus* ♂	Absent	–	Present	–	This paper
*R. prolixus* (Cojedes, Venezuela) ♀ x *R. nasutus* (Ceará, Brazil) ♂	Absent	–	Present (♂)^1^	–	24, 25
*R. prolixus* (Cundinamarca, Colombia) ♀ x *R. nasutus* (Ceará, Brazil) ♂	Absent	–	Present (♂)^1^	–	24, 25
*R. prolixus* (Honduras) ♀ x *R. nasutus* (Ceará, Brazil) ♂	Absent	–	Present (♂)^1^	–	24, 25
*R. prolixus* (Casanare, Colombia) ♀ x *R. nasutus* (Ceará, Brazil) ♂	Absent	–	Present (♂)^1^	Present^2^	24, 25
R. prolixus/R. robustus* (Boyaca, Colombia) ♀ x *R. nasutus* (Ceará, Brazil) ♂	Present	–	–	–	24, 25
*R. nasutus* ♀ x *R. prolixus* ♂	Absent		Present		This paper
*R. nasutus* (Ceará, Brazil) ♀ x *R. prolixus* (Cojedes, Venezuela) ♂	Present	–	–	–	24, 25
*R. nasutus* (Ceará, Brazil) ♀ x *R. prolixus* (Cundinamarca, Colombia) ♂	Present	–	–	–	24, 25
*R. nasutus* (Ceará, Brazil) ♀ x *R. prolixus* (Honduras) ♂	Present	–	–	–	24, 25
*R. nasutus* (Ceará, Brazil) ♀ x *R. prolixus* (Casanare, Colombia) ♂	Present	–	–	–	24, 25
*R. nasutus* (Ceará, Brazil) ♀ x R. prolixus/R. robustus* (Boyaca, Colombia) ♂	Present	–	–	–	24, 25
***R. robustus***					
*R. robustus* ♀ x *R. prolixus* ♂	Absent	–	–	Present^2^	This paper
*R. robustus* (Pará, Brazil) ♀ x *R. prolixus* (Amazonas, Brazil) ♂	Absent	–	Present (♀)^1^	–	24, 25
*R. robustus* (Pará, Brazil) ♀ x *R. prolixus* (Cojedes, Venezuela) ♂	Absent	–	Present	–	24, 25
*R. robustus* (Pará, Brazil) ♀ x *R. prolixus* (Cundinamarca, Colombia) ♂	Absent	–	Present^12^	–	24, 25
*R. robustus* (Pará, Brazil) ♀ x *R. prolixus* (Honduras) ♂	Absent	–	Present (♂)^1,2^	–	24, 25
*R. robustus* (Pará, Brazil) ♀ x *R. prolixus* (Casanare, Colombia) c	Absent	–	Present^2^	–	24, 25
*R. robustus* (Pará, Brazil) ♀ x R. prolixus/R. robustus* (Boyaca, Colombia) ♂	Absent	–	Present (♂)^1^	–	24, 25
*R. robustus* (Rondônia, Brazil) ♀ x *R. prolixus* (Cojedes, Venezuela) ♂	Absent	–	–	–	24, 25
*R. robustus* (Rondônia, Brazil) ♀ x *R. prolixus* (Cundinamarca, Colombia) ♂	Absent	–	–	–	24, 25
*R. robustus* (Rondônia, Brazil) ♀ x *R. prolixus* (Honduras) ♂	Absent	Present	–	–	24, 25
*R. robustus* (Rondônia, Brazil) ♀ x *R. prolixus* (Casanare, Colombia) ♂	Absent	–	Present (♀)^1,2^	–	24, 25
*R. robustus* (Rondônia, Brazil) ♀ x *R. prolixus* (Amazonas, Brazil) ♂	Absent	Present^2^	–	–	24, 25
*R. robustus* (Rondônia, Brazil) ♀ x *R. prolixus* (Pará, Brazil) ♂	Absent	–	Present	–	24, 25
*R. robustus* (Rondônia, Brazil) ♀ x R. prolixus/R. robustus* (Boyaca, Colombia) ♂	Absent	–	Present (♂)^1,2^	–	24, 25
*R. robustus* (Santander, Colombia) ♀ x *R. prolixus* (Cojedes, Venezuela) ♂	Absent	Absent	Absent	Absent	24, 25
*R. robustus* (Santander, Colombia) ♀ x *R. prolixus* (Cundinamarca, Colombia) ♂	Absent	Absent	Absent	Absent	24, 25
*R. robustus* (Santander, Colombia) ♀ x *R. prolixus* (Honduras) ♂	Absent	Absent	Absent	Absent	24, 25
*R. robustus* (Santander, Colombia) ♀ x *R. prolixus* (Casanare, Colombia) ♂	Absent	Absent	Absent	Absent	24, 25
*R. robustus* (Santander, Colombia) ♀ x *R. prolixus* (Amazonas, Brazil) ♂	Absent	Present	Present (♂)^1^	–	24, 25
*R. robustus* (Santander, Colombia) ♀ x *R. prolixus* (Pará, Brazil) ♂	Absent	Absent	Absent	Absent	24, 25
*R. robustus* (Santander, Colombia) ♀ x R. prolixus/R. robustus* (Boyaca, Colombia) ♂	Absent	Absent	Absent	Absent	24, 25
*R. robustus* (Lima, Peru) ♀ x *R. prolixus* (Cojedes, Venezuela) ♂	Absent	Absent	Absent	Absent	24, 25
*R. robustus* (Lima, Peru) ♀ x *R. prolixus* (Cundinamarca, Colombia) ♂	Absent	Absent	Absent	Absent	24, 25
*R. robustus* (Lima, Peru) ♀ x *R. prolixus* (Honduras) ♂	Absent	Absent	Absent	Absent	24, 25
*R. robustus* (Lima, Peru) ♀ x *R. prolixus* (Casanare, Colombia) ♂	Absent	Absent	Absent	Absent	24, 25
*R. robustus* (Lima, Peru) ♀ x *R. prolixus* (Amazonas, Brazil) ♂	Absent	Present	Present (♂)^1^	–	24, 25
*R. robustus* (Lima, Peru) ♀ x *R. prolixus* (Pará, Brazil) ♂	Absent	Absent	Absent	Absent	24, 25
*R. robustus* (Lima, Peru) ♀ x R. prolixus/R. robustus* (Boyaca, Colombia) ♂	Absent	Absent	Absent	Absent	24, 25
*R. robustus* (Mérida, Venezuela) ♀ x *R. prolixus* (Lara, Venezuela) ♂	Absent	Absent	Absent	Absent	26
*R. robustus* (Mérida, Venezuela) ♀ x *R. prolixus* (Guárico, Venezuela) ♂	Absent	Absent	Absent	Absent	26
*R. robustus* (Trujillo, Venezuela) ♀ x *R. prolixus* (Guárico, Venezuela) ♂	Absent	Absent	Absent	Absent	26
*R. robustus* (Trujillo, Venezuela) ♀ x *R. prolixus* (Lara, Venezuela) ♂	Absent	Absent	Absent	Absent	26
*R. robustus* (Venezuela) ♀ x *R. prolixus* (Venezuela) ♂	Absent	Absent	Absent	Absent	27
*R. prolixus* ♀ x *R. robustus* ♂	Absent	Absent	Absent	Absent	This paper
*R. prolixus* (Cojedes, Venezuela) x *R. robustus* (Santander, Colombia)	Absent	Absent	Absent	Absent	24, 25
* R. prolixus* (Cojedes, Venezuela) x *R. robustus* (Lima, Peru)	Absent	Absent	Absent	Absent	24, 25
* R. prolixus* (Cojedes, Venezuela) ♀ x *R. robustus* (Pará, Brazil) ♂	Absent	Present^2^	–	–	24, 25
* R. prolixus* (Cojedes, Venezuela) ♀ x *R. robustus* (Rondônia, Brazil) ♂	Absent	–	–	–	24, 25
* R. prolixus* (Cojedes, Venezuela) x R. prolixus/R. robustus* (Boyaca, Colombia)	Absent	Absent	Absent	Absent	24, 25
* R. prolixus* (Lara, Venezuela) ♀ x *R. robustus* (Mérida, Venezuela) ♂	Absent	Absent	Absent	Absent	26
* R. prolixus* (Lara, Venezuela) ♀ x *R. robustus* (Trujillo, Venezuela) ♂	Absent	Absent	Absent	Absent	26
* R. prolixus* (Guárico, Venezuela) ♀ x *R. robustus* (Mérida, Venezuela) ♂	Absent	Absent	Absent	Absent	26
* R. prolixus* (Guárico, Venezuela) ♀ x *R. robustus* (Trujillo, Venezuela) ♂	Absent	Absent	Absent	Absent	26
* R. prolixus* (Venezuela) ♀ x *R. robustus* (Venezuela) ♂	Present	–	–	–	27
* R. prolixus* (Cundinamarca, Colombia) x *R. robustus* (Santander, Colombia)	Absent	Absent	Absent	Absent	24, 25
* R. prolixus* (Cundinamarca, Colombia) x *R. robustus* (Lima, Peru)	Absent	Absent	Absent	Absent	24, 25
* R. prolixus* (Cundinamarca, Colombia) ♀ x *R. robustus* (Pará, Brazil) ♂	Absent	Present^2^	Present (♀)^1^	–	24, 25
* R. prolixus* (Cundinamarca, Colombia) ♀ x *R. robustus* (Rondônia, Brazil) ♂	Absent	–	–	–	24, 25
* R. prolixus* (Cundinamarca, Colombia) x R. prolixus/R. robustus* (Boyaca, Colombia)	Absent	Absent	Absent	Absent	24, 25
* R. prolixus* (Honduras) x *R. robustus* (Santander, Colombia)	Absent	Absent	Absent	Absent	24, 25
* R. prolixus* (Honduras) x *R. robustus* (Lima, Peru)	Absent	Absent	Absent	Absent	24, 25
* R. prolixus* (Honduras) ♀ x *R. robustus* (Rondônia, Brazil) ♂	Absent	–	–	–	24, 25
* R. prolixus* (Honduras) ♀ x *R. robustus* (Pará, Brazil) ♂	Absent	–	Present (♀)^1^	–	24, 25
* R. prolixus* (Honduras) ♂ x R. prolixus/R. robustus* (Boyaca, Colombia) ♀	Absent	Absent	Absent	Absent	24, 25
* R. prolixus* (Honduras) ♀ x R. prolixus/R. robustus* (Boyaca, Colombia) ♂	Absent	Absent	–	Present (♂)^1^	24, 25
* R. prolixus* (Casanare, Colombia) x *R. robustus* (Santander, Colombia)	Absent	Absent	Absent	Absent	24, 25
* R. prolixus* (Casanare, Colombia) x *R. robustus* (Lima, Peru)	Absent	Absent	Absent	Absent	24, 25
* R. prolixus* (Casanare, Colombia) ♀ x *R. robustus* (Pará, Brazil) ♂	Absent	–	Present (♀)^1^	–	24, 25
* R. prolixus* (Casanare, Colombia) ♀ x *R. robustus* (Rondônia, Brazil) ♂	Absent	–	Present	–	24, 25
* R. prolixus* (Amazonas, Brazil) ♀ x *R. robustus* (Pará, Brazil) ♂	Absent	–	Present (♂)^1^	–	24, 25
* R. prolixus* (Amazonas, Brazil) ♀ x *R. robustus* (Rondônia, Brazil) ♂	Absent	–	Present^2^	–	24, 25
* R. prolixus* (Amazonas, Brazil) ♀ x *R. robustus* (Santander, Colombia) ♂	Absent	–	Present^2^	–	24, 25
* R. prolixus* (Amazonas, Brazil) ♀ x *R. robustus* (Lima, Peru) ♂	Absent	–	Present (♂)^1^	–	24, 25
* R. prolixus* (Amazonas, Brazil) ♀ x R. prolixus/R. robustus* (Boyaca, Colombia) ♂	Absent	–	Present^2^	–	24, 25
* R. prolixus* (Amazonas, Brazil) ♂ x R. prolixus/R. robustus* (Boyaca, Colombia) ♀	Absent	Absent	Absent	Absent	24, 25
R. prolixus/R. robustus* (Boyaca, Colombia) x *R. robustus* (Santander, Colombia)	Absent	Absent	Absent	Absent	24, 25
R. prolixus/R. robustus* (Boyaca, Colombia) x *R. robustus* (Lima, Peru)	Absent	Absent	Absent	Absent	24, 25
R. prolixus/R. robustus* (Boyaca, Colombia) ♀ x *R. robustus* (Rondônia, Brazil) ♂	Absent	–	Present (♂)^1^	–	24, 25
R. prolixus/R. robustus* (Boyaca, Colombia) ♀ x *R. prolixus* (Amazonas, Brazil) ♂	Absent	Present^2^	–	–	24, 25
***R. neglectus***					
* R. neglectus* (Bahia, Brazil) ♀ x *R. prolixus* (Cojedes, Venezuela) ♂	Present	–	–	–	24, 25
* R. neglectus* (Bahia, Brazil) ♀ x *R. prolixus* (Cundinamarca, Colombia) ♂	Present	–	–	–	24, 25
* R. neglectus* (Bahia, Brazil) ♀ x *R. prolixus* (Honduras) ♂	Present	–	–	–	24, 25
* R. neglectus* (Bahia, Brazil) ♀ x *R. prolixus* (Casanare, Colombia) ♂	Present	–	–	–	24, 25
* R. neglectus* (Bahia, Brazil) ♀ x *R. prolixus* (Amazonas, Brazil) ♂	Absent	Absent	Absent	Absent	24, 25
* R. neglectus* (São Paulo, Brazil) ♀ x *R. prolixus* (São Paulo, Brazil) ♂	Present	–	–	–	43, 44, 45
* R. neglectus* (São Paulo, Brazil) ♀ x *R. prolixus* (Venezuela) ♂	Absent	Present	–	–	67
* R. neglectus* (São Paulo, Brazil) ♀ x *R. prolixus* (Cojedes, Venezuela) ♂	Present	–	–	–	24, 25
* R. neglectus* (São Paulo, Brazil) ♀ x *R. prolixus* (Cundinamarca, Colombia) ♂	Present	–	–	–	24, 25
* R. neglectus* (São Paulo, Brazil) ♀ x *R. prolixus* (Honduras) ♂	Present	–	–	–	24, 25
* R. neglectus* (São Paulo, Brazil) ♀ x *R. prolixus* (Casanare, Colombia) ♂	Present	–	–	–	24, 25
* R. neglectus* (São Paulo, Brazil) ♀ x *R. prolixus* (Amazonas, Brazil) ♂	Absent	Absent	Absent	Absent	24, 25
* R. neglectus* (Goiás, Brazil) ♀ x *R. prolixus* (Cojedes, Venezuela) ♂	Present	–	–	–	24, 25
* R. neglectus* (Goiás, Brazil) ♀ x *R. prolixus* (Cundinamarca, Colombia) ♂	Present	–	–	–	24, 25
* R. neglectus* (Goiás, Brazil) ♀ x *R. prolixus* (Honduras) ♂	Present	–	–	–	24, 25
* R. neglectus* (Goiás, Brazil) ♀ x *R. prolixus* (Casanare, Colombia) ♂	Present	–	–	–	24, 25
* R. neglectus* (Goiás, Brazil) ♀ x *R. prolixus* (Amazonas, Brazil) ♂	Absent		Present	–	24, 25
* R. neglectus* (Tocantins, Brazil) ♀ x *R. prolixus* (Cojedes, Venezuela) ♂	Present	–	–	–	24, 25
* R. neglectus* (Tocantins, Brazil) ♀ x *R. prolixus* (Cundinamarca, Colombia) ♂	Present	–	–	–	24, 25
* R. neglectus* (Tocantins, Brazil) ♀ x *R. prolixus* (Honduras) ♂	Present	–	–	–	24, 25
* R. neglectus* (Tocantins, Brazil) ♀ x *R. prolixus* (Casanare, Colombia) ♂	Present	–	–	–	24, 25
* R. neglectus* (Bahia, Brazil) ♀ x R. prolixus/R. robustus* (Boyaca, Colombia) ♂	Present	–	–	–	24, 25
* R. neglectus* (São Paulo, Brazil) ♀ x R. prolixus/R. robustus* (Boyaca, Colombia) ♂	Present	–	–	–	24, 25
* R. neglectus* (Goiás, Brazil) ♀ x R. prolixus/R. robustus* (Boyaca, Colombia) ♂	Present	–	–	–	24, 25
* R. neglectus* (Tocantins, Brazil) ♀ x R. prolixus/R. robustus* (Boyaca, Colombia) ♂	Present	–	–	–	24, 25
* R. prolixus* (São Paulo, Brazil) ♀ x *R. neglectus* (São Paulo, Brazil) ♂	Absent	Present	Present (♂)	–	43, 44, 45
* R. prolixus* (Venezuela) ♀ x *R. neglectus* (São Paulo, Brazil) ♂	Present	–	–	–	67
* R. prolixus* (Cojedes, Venezuela) ♀ x *R. neglectus* (Bahia, Brazil) ♂	Absent	–	Present (♂)^1^	Present^2^	24, 25
* R. prolixus* (Cojedes, Venezuela) ♀ x *R. neglectus* (São Paulo, Brazil) ♂	Absent	–	Present (♂)^1^	Present^2^	24, 25
* R. prolixus* (Cojedes, Venezuela) ♀ x *R. neglectus* (Goiás, Brazil) ♂	Absent	–	Present (♂)^1^	–	24, 25
* R. prolixus* (Cojedes, Venezuela) ♀ x *R. neglectus* (Tocantins, Brazil) ♂	Absent	–	Present (♂)^1^	–	24, 25
* R. prolixus* (Cundinamarca, Colombia) ♀ x *R. neglectus* (Bahia, Brazil) ♂	Absent	–	Present (♂)^1^	–	24, 25
* R. prolixus* (Cundinamarca, Colombia) ♀ x *R. neglectus* (São Paulo, Brazil) ♂	Absent	–	Present (♂)^1^	–	24, 25
* R. prolixus* (Cundinamarca, Colombia) ♀ x *R. neglectus* (Goiás, Brazil) ♂	Absent	–	Present (♂)^1^	–	24, 25
* R. prolixus* (Cundinamarca, Colombia) ♀ x *R. neglectus* (Tocantins, Brazil) ♂	Absent	–	Present (♂)^1^	–	24, 25
* R. prolixus* (Honduras) ♀ x *R. neglectus* (Bahia, Brazil) ♂	Absent	–	Present (♂)^1^	Present^2^	24, 25
* R. prolixus* (Honduras) ♀ x *R. neglectus* (São Paulo, Brazil) ♂	Absent	–	Present (♂)^1^	Present^2^	24, 25
* R. prolixus* (Honduras) ♀ x *R. neglectus* (Goiás, Brazil) ♂	Absent	–	Present (♂)^1^	–	24, 25
* R. prolixus* (Honduras) ♀ x *R. neglectus* (Tocantins, Brazil) ♂	Absent	–	Present (♂)^1^	–	24, 25
* R. prolixus* (Casanare, Colombia) ♀ x *R. neglectus* (Bahia, Brazil) ♂	Present	–	–	–	24, 25
* R. prolixus* (Casanare, Colombia) ♀ x *R. neglectus* (São Paulo, Brazil) ♂	Present	–	–	–	24, 25
* R. prolixus* (Casanare, Colombia) ♀ x *R. neglectus* (Goiás, Brazil) ♂	Present	–	–	–	24, 25
* R. prolixus* (Casanare, Colombia) ♀ x *R. neglectus* (Tocantins, Brazil) ♂	Absent	–	Present (♂)^1^	–	24, 25
* R. prolixus* (Amazonas, Brazil) ♀ x *R. neglectus* (São Paulo, Brazil) ♂	Absent	–	Present (♂)^1^	–	24, 25
* R. prolixus* (Amazonas, Brazil) ♀ x *R. neglectus* (Bahia, Brazil) ♂	Absent	–	Present (♂)^1^	–	24, 25
* R. prolixus* (Amazonas, Brazil) ♀ x *R. neglectus* (Goiás, Brazil) ♂	Absent	–	Present (♂)^1^	–	24, 25
* R. prolixus* (Amazonas, Brazil) ♀ x *R. neglectus* (Tocantins, Brazil) ♂	Absent	–	Present (♂)^1^	–	24, 25
R. prolixus/R. robustus* (Boyaca, Colombia) ♀ x *R. neglectus* (São Paulo, Brazil) ♂	Present	–	–	–	24, 25
R. prolixus/R. robustus* (Boyaca, Colombia) ♀ x *R. neglectus* (Bahia, Brazil) ♂	Present	–	–	–	24, 25
R. prolixus/R. robustus* (Boyaca, Colombia) ♀ x *R. neglectus* (Goiás, Brazil) ♂	Absent	–	Present (♂)^1^	–	24, 25
R. prolixus/R. robustus* (Boyaca, Colombia) ♀ x *R. neglectus* (Tocantins, Brazil) ♂	Absent	–	Present (♂)^1^	–	24, 25
**Intraspecific crosses**					
* R. prolixus* (Cojedes, Venezuela) x *R. prolixus* (Cundinamarca, Colombia)	Absent	Absent	Absent	Absent	24, 25
* R. prolixus* (Cojedes, Venezuela) ♂ x *R. prolixus* (Honduras) ♀	Absent	Absent	Absent	Absent	24, 25
* R. prolixus* (Cojedes, Venezuela) ♀ x *R. prolixus* (Honduras) ♂	Absent	Absent	–	Present (♀)^1,2^	24, 25
* R. prolixus* (Cojedes, Venezuela) x *R. prolixus* (Casanare, Colombia)	Absent	Absent	Absent	Absent	24, 25
* R. prolixus* (Cojedes, Venezuela) ♀ x *R. prolixus* (Amazonas, Brazil) ♂	Absent	Present	–	–	24, 25
* R. prolixus* (Cundinamarca, Colombia) ♂ x *R. prolixus* (Honduras) ♀	Absent	Absent	Absent	Absent	24, 25
* R. prolixus* (Cundinamarca, Colombia) ♀ x *R. prolixus* (Honduras) ♂	Absent	Absent	–	Present (♀)^1,2^	24, 25
* R. prolixus* (Cundinamarca, Colombia) x *R. prolixus* (Casanare, Colombia)	Absent	Absent	Absent	Absent	24, 25
* R. prolixus* (Cundinamarca, Colombia) ♀ x *R. prolixus* (Amazonas, Brazil) ♂	Absent	Present	–	–	24, 25
* R. prolixus* (Honduras) x *R. prolixus* (Casanare, Colombia)	Absent	Absent	Absent	Absent	24, 25
* R. prolixus* (Honduras) ♀ x *R. prolixus* (Amazonas, Brazil) ♂	Absent	Present^2^	–	–	24, 25
* R. prolixus* (Casanare, Colombia) x *R. prolixus* (Amazonas, Brazil)	Absent	Present^2^	Present	–	24, 25
* R. prolixus* (Amazonas, Brazil) ♀ x *R. prolixus* (Cojedes, Venezuela) ♂	Absent	Absent	Absent	Absent	24, 25
* R. prolixus* (Amazonas, Brazil) ♀ x *R. prolixus* (Cundinamarca, Colombia) ♂	Absent	Absent	Absent	Absent	24, 25
* R. prolixus* (Amazonas, Brazil) ♀ x *R. prolixus* (Honduras) ♂	Absent	Absent	Absent	Absent	24, 25
* R. prolixus* (Amazonas, Brazil) ♀ x *R. prolixus* (Casanare, Colombia) ♂	Absent	–	Present	–	24, 25

*R. prolixus according to collectors and *R. robustus* according to chromatic characters [24]; ^1^partial sterility; ^2^backcrosses; ♀: female; ♂: male.

Recently, Filée et al. [13], using molecular taxonomy, confirmed the presence of *R. prolixus* in Brazil (Pará state) (specimens morphologically identified as *R. robustus*). Given this and, above all, of the potential distribution of *R. prolixus* that covers southern Brazil, at the border between Brazil, Peru, Colombia, southern Mexico, Guatemala, El Salvador, and Honduras [55], we emphasize the need for phylogeographic studies with field specimens, covering all potential distribution areas of the species and combining different taxonomic tools [13,19,56], especially given the vectorial importance of *R. prolixus* for the transmission of CD [10].

Given all these problems involving the distribution of *R. prolixus* [13,19,38,48–55], we used, for the first time, specimens that had their specific status confirmed by phylogenomic analyzes, that is, phenotypically and genotypically characterized as *R. prolixus* [13], ensuring that the barriers characterized here really are from crosses between *R. prolixus* and other *Rhodnius* species (Table 1 and 2). With the exception of the cross between *R. prolixus* females and *R. robustus* males that produced hybrids up to the F5, demonstrating that, under laboratory conditions, no reproductive barriers were detected, all other combinations produced hybrids that became unviable by postzygotic barriers (inviability, sterility and/or hybrid collapse [30,32,33]).

The barrier present between *R. neivai* females and *R. prolixus* males and between *R. robustus* females and *R. prolixus* males was hybrid collapse (or hybrid breakdown), once the F1 and F2 hybrids were viable and fertile, while F3 were sterile (Table 1 and 2). This barrier has already been characterized in the genera *Triatoma* [31] and *Mepraia* Mazza, Gajardo & Jörg, 1940 [57], but represents first formal record in the genus *Rhodnius*, because although hybrid mortality in backcrosses was observed by Barrett [24,25] (Table 2), the authors do not indicate which evolutionary events were related to the hybrids lineage breakdown.

**Fig 2:**
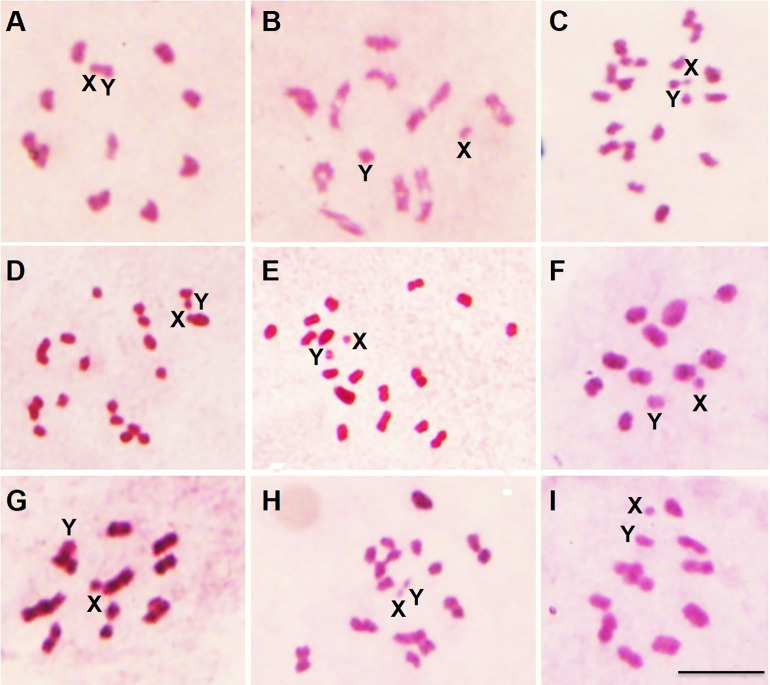
Prometaphases and metaphases of hybrids: from the cross between *R. neivai* ♀ x *R. prolixus* ♂ of first (A), second (B) and third (C) generation; from the cross between *R. nasutus* ♀ x *R. prolixus* ♂ (D) and *R. prolixus* ♀ x *R. nasutus* ♂ (E) of first generation; from the cross between *R. robustus* ♀ x *R. prolixus* ♂ of first (F), second (G) and third (H) generation; and from the cross between *R. prolixus* ♀ x *R. robustus* ♂ of fifth generation (I). A, B, F, G, I: Note that 100% of the chromosomes were paired. C, D, E, H: Note pairing errors between different autosomes. X: X sex chromosome; Y: Y sex chromosome; Bar: 10 µm.

Cytogenetic studies on the gonads of these hybrids demonstrated that the chromosomes of the F1 (Fig 2A and 2F) and F2 hybrids (Fig 2B and 2G) presented 100% pairing, while the F3 hybrids presented some monovalent chromosomes resulting from non-pairing between homologues (Fig 2C and 2H), which result in genetically imbalanced gametes (inviable) and, consequently, infertility in the interspecific hybrids [55] – confirming the 0% hatch rate of F3 x F3 intercrosses (Table 1).

Crosses between *R. prolixus* and *R. robustus* have already been performed by several authors (Table 2). In the direction in which we observed the hybrid collapse (*R. robustus* females and *R. prolixus* males), the results observed by other authors were very diverse – ranging from total absence of barriers, as well as postzygotic isolation (inviability and/or hybrid sterility) [24–27]. The absence of reproductive barriers under laboratory conditions – as we observed for the other direction of the cross: *R. prolixus* females and *R. robustus* males which produced hybrids up to F5 (Table 1) and all offspring were fertile and without chromosomal pairing errors (Fig 2I) – does not allow taxonomic conclusions to be proposed, since possible prezygotic barriers, such as temporal, ecological and habitat [0, 32, 33], are disregarded. However, in the last decade data from experimental crosses were combined with phylogenetic systematics, and the synonymization of *R. taquarussuensis* Rosa et al., 2017 and *R. milesi* Carcavallo, Rocha, Galvão & Jurberg, 2001 with *R. neglectus* was proposed [6,58]. It is worth noting that the absence of reproductive barriers was not used as support for the taxonomic changes, but rather the biological data extracted from the crosses, as F1 hatching and mortality rates, that were very close between parents and offspring.

*Rhodnius robustus* represents a paraphyletic complex of species [15,59]. Initial studies indicated the presence of four cryptic lineages [15]. Currently, at least five lineages are recognized [60] and some of them have been described as valid species, namely, *R. montenegrensis* [61] and *R. marabaensis* [62]. Therefore, the different barriers observed between *R. prolixus* and *R. robustus* (Table 2) may come from the different lineages used: specimens from Pará, Brazil [24,25], for example, may be *R. marabaensis* [63] and those from Rondônia, Brazil [24,25] may be *R. montenegrensis* [63] – regardless of the direction and origin of *R. prolixus*, most of the crossings with all insects from Pará and from Rondônia showed reproductive isolation (demonstrating that these taxa represent different species [30,32,33]).

The characterization of one or more barriers in one of the crossing directions (partial reproductive isolation) is already sufficient to confirm the specific status of *R. prolixus* and *R. robustus* (or *R. marabaensis* and/or *R. montenegrensis*) (Table 2). Filée et al. [13] analyzed the phylogenetic position of *R. robustus* specimens from the same colony that we used in the crosses and demonstrated a mito-nuclear conflict, since they suggested introgression between *R. montenegrensis* and *R. prolixus* with this population of *R. robustus*. Furthermore, mitochondrial markers suggest that this population of *R. robustus* represents *R. montenegrensis* [13]. Regardless of whether it is *R. robustus* or *R. montenegrensis*, our results do not rule out the possibility that introgression has occurred/is occurring under natural conditions between this taxon and *R. prolixus* (mainly because the distribution area of *R. montenegrensis* is expanding to other Latin American countries [64,65]), since hybrids were viable and fertile in both directions (up to F2 in the direction of *R. robustus* females with *R. prolixus* males and up to F5 in the direction of *R. prolixus* females with *R. robustus* males) (Table 1), which facilitates the occurrence of backcrossing and exchange of interspecific genetic material.

*Rhodnius nasutus*, when crossed with *R. prolixus*, produced completely infertile hybrids (Table 1) that presented several chromosome pairing errors (Fig 2D and 2E) (and, consequently, inviable gametes [66]), characterizing the hybrid sterility observed in Table 2. Other crosses had already been performed between these species (using specimens from Ceará, Brazil [24,25]) and most crosses with females of *R. nasutus* presented prezygotic barriers and, with males, postzygotic barriers (Table 2). Similarly, all crosses between *R. neglectus* and *R. prolixus* [24,25,43–45,67] demonstrated similar results: most crosses with females of *R. neglectus* presented prezygotic barriers and, with males, postzygotic barriers. In addition, when *R. pictipes* was crossed with *R. prolixus* [68], nymphs hatched but died before reaching adulthood (hybrid inviability) (Table 2). Thus, unlike what was observed for *R. prolixus* and *R. robustus*, the absence of adult hybrids or the sterility of these organisms prevent possible introgression events between these species and *R. prolixus*.

Finally, some intraspecific crosses allowed the characterization of reproductive barriers (Table 2), most of them between *R. prolixus* from Amazonas, Brazil and other allopatric populations of the species (Honduras, Venezuela and Colombia). This fact is intriguing, as it is at odds with the hegemonic biological species concept (“group of actually or potentially interbreeding natural populations which are reproductively isolated from other such groups [69]”), highlighting that the parents used represent different species. In the state of Amazonas, six species of *Rhodnius* have been reported (*R. amazonicus* Almeida, Santos & Sposina, 1973, *R. brethesi* Matta, 1919, *R. montenegrensis*, *R. paraensis* Sherlock, Guitton & Miles, 1977, *R. pictipes* and *R. robustus*) [63]. Considering the phylogenetic proximity [13], the morphological relationship [18] and, above all, the “intraspecific” reproductive isolation, we believe that the Amazon specimens used by Barrett [25,26] were *R. robustus* or *R. montenegrensis*.

## Conclusions

This study demonstrates that *R. nasutus*, *R. neivai*, and *R. robustus* are reproductively isolated from *R. prolixus* in at least one direction, confirming the specific status of the four taxa. Based on the observed reproductive barriers, we propose that there is no possibility of introgression between *R. prolixus* and *R. nasutus*, in contrast to *R. neivai* and *R. robustus*, which potentially exchange genetic material with *R. prolixus* through introgression under natural conditions. Finally, we synthesized all the literature data related to intra- and interspecific crosses of *R. prolixus*, demonstrating that *R. pictipes* and *R. neglectus* are also reproductively isolated from *R. prolixus*. In addition, our findings also to drawing attention to the reproductive isolation observed between allopatric populations of *R. prolixus*, we emphasize the necessity for an extensive phylogenomic investigation involving field-collected specimens across the full geographical range of *R. prolixus*, in order to elucidate the taxonomic complexities presented about this species of great importance for the epidemiology of CD.
